# An Unusual First Manifestation of Hodgkin Lymphoma: Epitrochlear Lymph Node İnvolvement—A Case Report and Brief Review of Literature

**DOI:** 10.1177/2324709617706709

**Published:** 2017-04-27

**Authors:** Veysi Hakan Yardimci, Aytul Hande Yardimci

**Affiliations:** 1 Acibadem Hospital, Istanbul, Turkey; 2 Istanbul Training and Research Hospital, Istanbul, Turkey

**Keywords:** epitrochlear lymph nodes, Hodgkin lymphoma, lymphadenopathy, elbow

## Abstract

Although epitrochlear lymph nodes may be enlarged as a part of generalized lymphadenopathy, isolated enlargement of epitrochlear lymph nodes is rarely seen. We describe Hodgkin’s lymphoma in a 55-year-old male who presented with isolated epitrochlear lymphadenopathy of his right arm. In the histopathological examination of the epitrochlear lymph node was a lymphocyte-rich Hodgkin lymphoma with a clinical grade (CS IA) diagnosed. The diagnosis was confirmed, via the bone marrow biopsy and positron emission tomography/computed tomography imaging, as pathological stage PS IA and clinical stage CS IA. Epitrochlear lymph node involvement, as a first presentation, is rarely seen in Hodgkin’s lymphoma. The aim of this study was to recapitulate the possible background diseases arising on the basis of an asymptomatic epitrochlear lymphadenopathy, to review the Hodgkin lymphoma presenting with primary epitrochlear lymphadenopathy in light of the literature, and to highlight the importance of a careful examination of the elbow site in routine physical examination.

## Introduction

Epitrochlear region lymphadenopathies are observed in forearm and hand pathologies. Epitrochlear lymph nodes, which are nonpalpable normally, generally become palpable as a result of a pathological disease.^1^

Epitrochlear lymphadenopathy is frequently a component of generalized lymphadenopathy; however, it may be manifested as an isolated form. Cat scratch, leprosy, leishmaniosis, tuberculosis, and filariasis are rare and benign causes of isolated epitrochlear lymphadenopathy.^1^ Lymphoma and malignant melanoma may be counted as malignant involvements. Epitrochlear lymph node involvement is a very rarely detected finding in both Hodgkin and non-Hodgkin lymphoma.

## Case Report

**Figure 1.**
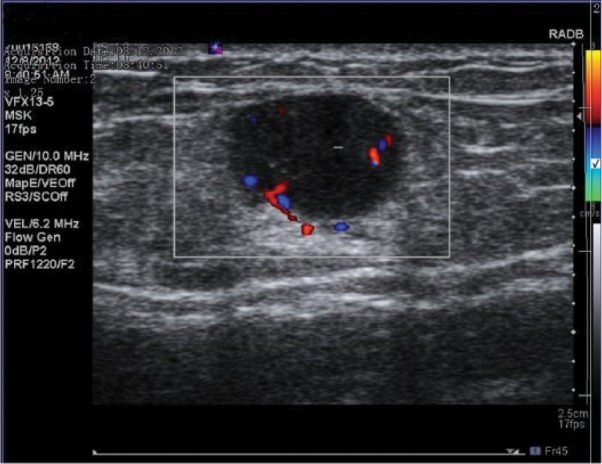
Doppler US image demonstrates an oval, hypoechoic, inhomogeneous epitrochlear lymph node with regular margin and peripheral flow signals.

A 55-year-old-male patient presented with a complaint of painful edema at the elbow level of the right arm, which had been persisting for almost a month. A 2-cm sized solid, medium hard, palpable mass was detected in the medial aspect of the right forearm in the physical examination. No finding of redness, temperature, rise or infection was detected. Ultrasound (US) examination revealed a 16 × 12 × 10 mm sized, ill-defined, oval and hypoechoic lymph node without hilar hyperechoic structure. Doppler US examination has detected a peripheral (capsular) flow signals (Figure 1). No significant abnormal value was determined in the blood count. The lymphocyte/neutrophil ratio and the leukocyte count were within normal ranges. Excisional biopsy of epitrochlear lymph node was performed under general anesthesia.

**Figure 2.**
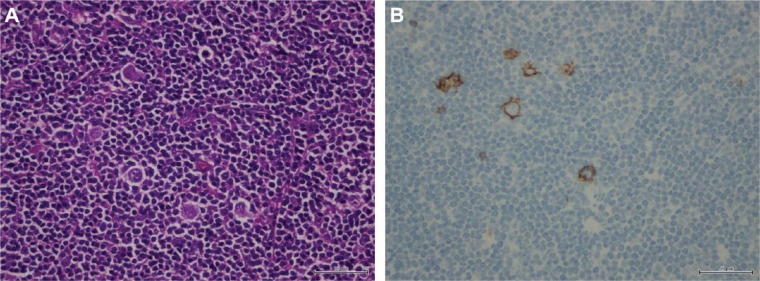
Microscopic findings of excisional biopsy specimen obtained from right epitrochlear lymph node: (A) Reed-Sternberg cell with lymphocyte-rich background was noted (hematoxylin-eosin, 40×); (b) CD15 positivity on immunohistochemistry for RS cells (40×).

Histopathological examination revealed lymphoid follicles (CD23) with significant germinal centers and significantly widened mantle zones in the lymph node. The neoplastic cells were DC30+, CD15+, CD20−, CD3−, CD5−, CD10−, CD23−, IGD−, and EBV−. Ki67 proliferation indicator was frequently positively stained (Figure 2).

The pathological diagnosis was classic-type Hodgkin lymphoma, lymphocyte-rich type (clinical stage CS IA, pathological stage PS IA).

The preoperative anamnesis of the patient was as follows: HIV negative, lymphoma negative family history, and other risk factors except that the age decade were negative. No additional palpable lymph node was detected on the physical examination; no clinical B symptoms such as fever and weight loss within a short period with an unknown reason; and no night sweating, itching, cough, respiratory distress, or chest pain was detected. No hepatosplenomegaly was determined.

In the histopathological examination of the bone marrow biopsy, no finding in favor of lymphoma was detected. The results were compatible with normocellular bone marrow. The postoperative blood tests, alanine aminotransferase, aspartate aminotransferase, lactate dehydrogenase, C-reactive protein, protein electrophoresis, erythrocyte sedimentation rate, and fibrinogen values were within normal ranges.

**Figure 3.**
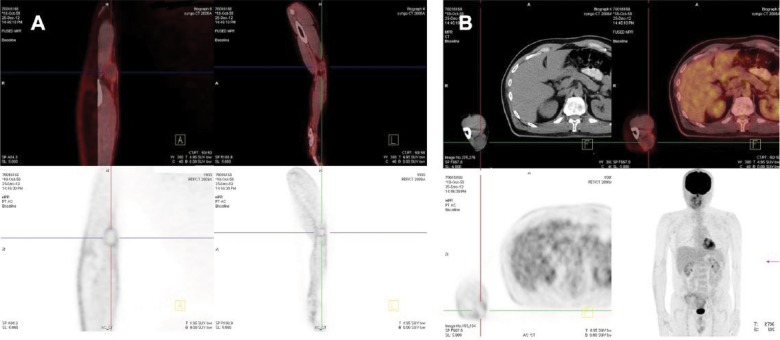
Follow-up FDG PET scan (axial, coronal, sagittal views): (a) There is moderate uptake of tracer in the surgical area of the right epitrochlear region and (b) normal uptake of FDG in the whole body.

The patient, who underwent clinical staging, was examined by FDG (flourodeoxyglucose uptake) positron emission tomography/computed tomography (PET-CT) imaging. PET-CT scan demonstrated a slightly increased FDG uptake at the region of the epitrochlear lymph node of the right arm. No pathological uptake except the right epitrochlear lymph node region was detected (Figure 3).

The patient underwent 2 courses of chemotherapy (ABVD protocol) plus radiotherapy with the diagnosis of lymphocyte-rich-type Hodgkin lymphoma (CS IA). The patient is being followed-up within a 4-year control interval without any complication.

## Discussion

The complicated surgical anatomy of the mid-arm lymph nodes was first described by Fujiwara et al.^2^ Epitrochlear lymph nodes are found 2 to 3 cm above the medial epicondyle of the humerus, on the biceps fascia. They are frequently 1 or 2 in number, but may sometimes be 3 and very rarely 4 in number.^3,4^

Generally, the superficial lymphatic systems of the middle finger, ring finger, and the small finger and medial sides of the anterior and the posterior arm–forearm are drained into the epitrochlear lymph nodes.^3-5^ They are united with the deep lymphatics and other superficial lymphatics, and they drain into the lateral axillary lymph nodes.

Physical examination, as well as the anamnesis of the patient, routine blood tests, and US examination are important in the investigation of masses within the elbow region.^4^

In the study by Selby et al, there was no palpable epitrochlear lymphadenopathy detected in the physical examination of 140 healthy patients. But palpable epitrochlear nodes were present in 27% of the 184 patients with diseases in which lymphadenopathy occurs.^6^

This result highlights the importance of routine epitrochlear region examination in patients who are lymphadenopathy-positive for any site in the body.

Following the physical examination of the superficial lymph nodes of the arm, ultrasonography examination is more effective and a cheaper radiological imaging technique than other modalities. This imaging method includes morphological structural characteristics, number, longitudinal-transvers diameters, shape, and borders of the lymph node. Vascularity is evaluated by Doppler US examination.^4^

The masses of the elbow region can be of nodal or extranodal origin. Nodal masses are acute lymphadenitis (cutaneous infections, cat scratch disease), tubercular lymphadenitis, sarcoidosis-related lenfadenitis, lymphadenitis due to foreign bodies or IV drug abuse, lymphomas, and metastatic lymphadenopathies. Extranodal masses are tumors (median nerve tumors, fibromas, hemangiomas, lipomas, Merkel cell tumors), sebaceous cysts, abscesses (elbow joint septic arthritis), Kimura disease, and cutaneous or subcutaneous hematogenous metastases.^2,4^

In the literature, very limited information is available on patients with Hodgkin disease with epitrochlear lymph node involvement.^7^ This very rare presentation of Hodgkin disease was first described 1932 by Rouviere.^8^ In the subsequent years, authors have reported epitrochlear lymph node positivity in Hodgkin disease with physical examination findings in rare isolated cases or several large series.

Weiss and Jenkins have determined epitrochlear or popliteal lymph node involvement in only 4 of 149 patients with Hodgkin disease (2.6%); however, involvements of the lymph nodes of other sites were concomitant in all cases.^8^

Kaplan has reported a 50% rate of axillary lymph node involvement in his series including 340 patients; however, there was epitrochlear lymph node involvement in only 3 cases (0.88%).^9^ In the series of Kaplan, other lymph node involvement was positive and the disease was not stage I in any of the 3 cases.

Weiss and Jenkins have reported a case with only epitrochlear lymph node involvement of Hodgkin lymphoma clinical stage CS IA. However, staging laparotomy revealed a small, single Hodgkin disease nodule in the spleen.^8^ In the long-term (30 years) study of Chang et al, 1180 clinical stage CS IA-IIB Hodgkin patients were evaluated. They detected only 11 (1%) patients with epitrochlear lymphadenopathy positivity.^7^

In conclusion, palpable epitrochlear lymph nodes are not seen frequent in clinical practice. In light of the aforementioned information, examination of the elbow lymph nodes as a part of the routine physical examination may play a key role in the diagnoses of malignant and benign diseases.

Early pathological diagnosis of Hodgkin disease through physical examination and radiological imaging of a superficial lymph node when no other clinical or laboratory finding is present may be the route for cure of this malignant disease.
